# Functional Tuning of Intrinsically Disordered Regions in Human Proteins by Composition Bias

**DOI:** 10.3390/biom12101486

**Published:** 2022-10-15

**Authors:** Kristina Kastano, Pablo Mier, Zsuzsanna Dosztányi, Vasilis J. Promponas, Miguel A. Andrade-Navarro

**Affiliations:** 1 Institute of Organismic and Molecular Evolution, Faculty of Biology, Johannes Gutenberg University, Biozentrum I, Hans-Dieter-Hüsch-Weg 15, 55128 Mainz, Germany; 2 Department of Biochemistry, ELTE Eötvös Loránd University, Pázmány Péter stny 1/c, H-1117 Budapest, Hungary; 3 Bioinformatics Research Laboratory, Department of Biological Sciences, University of Cyprus, 1678 Nicosia, Cyprus

**Keywords:** compositionally biased regions, low complexity regions, intrinsically disordered regions, liquid–liquid phase separation

## Abstract

Intrinsically disordered regions (IDRs) in protein sequences are flexible, have low structural constraints and as a result have faster rates of evolution. This lack of evolutionary conservation greatly limits the use of sequence homology for the classification and functional assessment of IDRs, as opposed to globular domains. The study of IDRs requires other properties for their classification and functional prediction. While composition bias is not a necessary property of IDRs, compositionally biased regions (CBRs) have been noted as frequent part of IDRs. We hypothesized that to characterize IDRs, it could be helpful to study their overlap with particular types of CBRs. Here, we evaluate this overlap in the human proteome. A total of 2/3 of residues in IDRs overlap CBRs. Considering CBRs enriched in one type of amino acid, we can distinguish CBRs that tend to be fully included within long IDRs (R, H, N, D, P, G), from those that partially overlap shorter IDRs (S, E, K, T), and others that tend to overlap IDR terminals (Q, A). CBRs overlap more often IDRs in nuclear proteins and in proteins involved in liquid-liquid phase separation (LLPS). Study of protein interaction networks reveals the enrichment of CBRs in IDRs by tandem repetition of short linear motifs (rich in S or P), and the existence of E-rich polar regions that could support specific protein interactions with non-specific interactions. Our results open ways to pin down the function of IDRs from their partial compositional biases.

## 1. Introduction

Many proteins are found to have intrinsically disordered regions (IDRs) and it has been proposed that their flexible properties are fundamental in their role as scaffolds for protein interactions [1]. Given their exposure to the solvent and flexibility, IDRs are easily subjected to extensive post-translational modifications that regulate their interactions [2].

Precisely because of their lack of structural constraints, IDRs have faster rates of evolution compared to globular domains [3]. IDRs can be found with short linear motifs (SLiMs) for post-translational modifications and interactions, often present in tandem and dynamically created and destroyed, which further provides an evolutionary advantage [4]. As a result, sequence homology has limited use to assess the function of IDRs.

In search of sequence properties to characterize IDRs alternative to sequence homology and SLiMs we found that low complexity could be a good candidate. While most protein sequences are composed of a variety of amino acid types, many proteins have sequence regions displaying a reduced number of different amino acids known as compositionally biased regions (CBRs [5]). In human proteins these regions have been estimated to be present in 44% of proteins, together covering 15% of total sequence [6].

The low complexity properties of IDRs have been noted [7] and CBRs have been utilized as a feature for IDR prediction [8]. However, low complexity is not a necessary property of IDRs [9], which can be more effectively detected by studying the physicochemical properties of consecutive amino acids [9].

While CBRs have a tendency to adopt flexible structures different to regions with average amino acid composition, which tend to form globular structures facilitated by the alternance of amino acid properties that results in the formation of elements of secondary structure [10], CBRs can also adopt structures. For example, glutamine homorepeats (polyQ) have been found to adopt alpha-helical structure [11]; this is dependent on the context of the sequence and polyQ can adopt other structures (see, e.g., [12]). In addition, it has been noted that many CBRs display periodicities and it was hypothesized that these could facilitate the formation of structure [9,13]. In fact, regions containing only one (polyX) or two (polyXY) amino acids within the IDRs of human proteins can be assigned experimental structural information more often than more complex IDR fragments [14]. On the other hand, protein tandem repeats, which are frequently found in protein sequences [15,16], are similar to CBRs when they become very short [9], and while they can form structures, it was found that when such protein tandem repeats are very perfect, they are more unstructured [17].

Our hypothesis is that by studying the overlap of CBRs to predicted IDRs in all human proteins we will be able to find properties of CBRs allowing IDRs to gain structure and function in particular contexts, for example upon protein binding [18]. With this goal in mind, we explore the overlap of CBRs, here defined as protein regions with at least one enriched amino acid, and IDRs in terms of extent, position and use in the cellular context, considering the enriched amino acid of the CBR (type).

Our results define the differential association of particular types of CBRs within IDRs and suggest the study of composition bias as a promising approach to the functional characterization of IDRs.

## 2. Results and Discussion

In order to characterize the compositional biases of IDRs in human proteins, we first evaluated the overlap between CBRs and IDRs for different types of CBRs. For this, we computed CBRs and IDRs for all human proteins using two well accepted methods: CAST [5] and IUPred2a [19], with default parameters, as well as the disorder predictions made with MobiDB-lite that are a consensus from many prediction tools [20] and can be downloaded from the MobiDB database [21] (see Methods for details). We observed a total of 18,222 CBRs, considering as CBRs regions enriched in one specific amino acid each time (see Methods for details), in 9080 proteins out of the 20,609 human proteins (see Supplementary File S1). These CBRs have a median length of 73 and cover 2.2 M residues. With IUPred we found 22,453 IDRs in 9084 proteins, with a median length of 53 and covering 1.9 M residues. With MobiDB-lite we found 26,349 IDRs in 11,331 proteins, with a median length of 39 and covering 1.6 M residues. The results of the following analyses that were produced with both IUPred and MobiDB-lite are similar, so we will present those of IUPred for the rest of the manuscript for simplicity, except when we explicitly mention otherwise.

**Figure 1:**
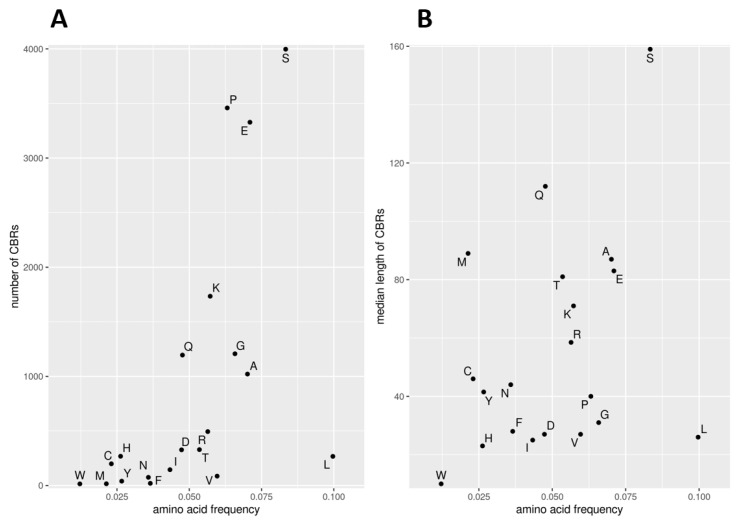
Properties of CBRs by type versus amino acid frequency. (**A**) Numbers of CBRs versus amino acid frequency. (**B**) Median CBR length (residues) versus amino acid frequency.

We found CBRs from all 20 amino acids in the human proteome. The numbers of CBRs by type are very variable ranging from 3997 to just 15 (for S and W, respectively; Table 1). Their numbers correlate somewhat with amino acid frequency (coefficient of determination R^2^ = 0.31). Indeed, W is both the least abundant amino acid and the one with fewer CBRs; CBRs of most amino acids with frequency below 0.06 have fewer than 500 occurrences (Figure 1A). However, there are outliers. The most abundant amino acid, L (0.10), has just 267 CBRs. The second most abundant, S (0.083), has the most occurrences (3997). CBRs of hydrophobic residues I, L and V have low occurrence (below 500) and an almost identical low mean length (around 26 residues; Figure 1B), despite their very different amino acid frequencies. CBRs of A, which is also hydrophobic but has a smaller size, occur more often (1021 times) and have longer median length (87).

The median length of CBRs is also very variable and correlates worse with amino acid frequency than number of CBRs (Figure 1B; coefficient of determination R^2^ = 0.11). W, the less abundant amino acid, produces the shortest CBRs with a median length of 10 residues. S, which contributed to the most abundant CBRs, stands out even more as making the longest CBRs with median length of 159 residues.

No clear correlation can be found between the frequency of CBRs and their length: see for example P, which makes the 2nd most abundant CBRs but results in average lengths, or M, which is extremely infrequent (16 occurrences) but makes the third longest CBRs. Together, these results support that CBRs are not just randomly occurring due to background amino acid frequencies, and that amino acid properties such as low hydrophobicity and small size may favor their contribution to CBRs.

### 2.1. Amount of Overlap by CBR-Type

**Figure 2:**
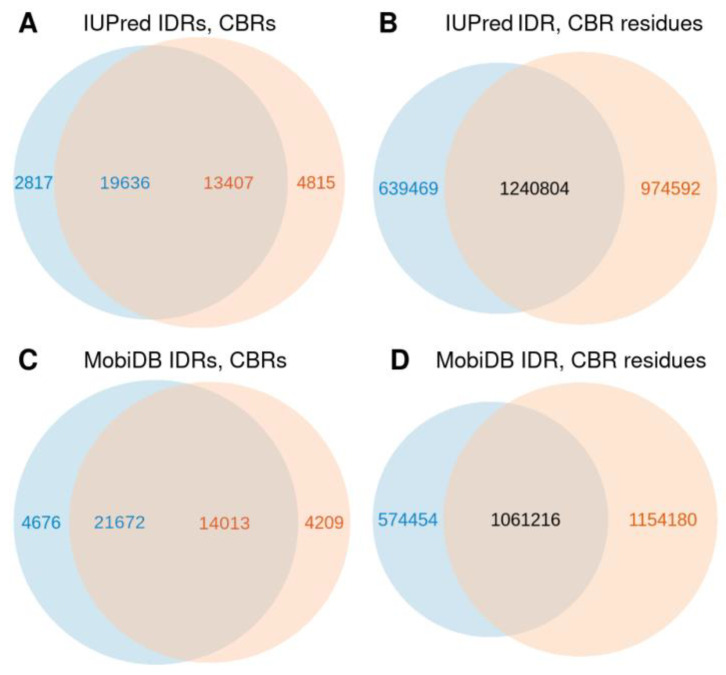
Overlap between CBRs and IDRs described by Venn diagrams. (**A**) By regions with IUPred predictions. (**B**) By residues with IUPred predictions. (**C**) By regions with MobiDB-lite predictions. (**D**) By residues with MobiDB-lite predictions. A given residue could be in multiple CBRs because CBRs of different types might overlap, but such residues were only counted once. IDRs do not overlap each other by definition. Note that the numbers of CBRs and IDRs overlapping (in **A** or in **C**) are different because a region of one type may overlap multiple regions of the other type.

The majority of CBRs overlap IDRs and vice versa (74% of CBRs and 87% of IDRs, for the IUPred predictions, Figure 2A; 77% and 82%, respectively for the MobiDB-lite predictions, Figure 2C) supporting the idea that composition bias and disorder are properties of protein sequences that, while not being identical, are strongly related. There are more IDRs predicted than CBRs, but IDRs are slightly shorter and thus the ratio of IDRs overlapping CBRs is higher than one (19,636/13,407 = 1.5) because multiple short IDRs may overlap a longer CBR.

In terms of residues, more than half of the residues in CBRs and two thirds of residues in IDRs overlap (56% and 66%, respectively, Figure 2B; 49% and 65%, respectively, Figure 2D). This suggests that residues in IDRs have a tendency to be within CBRs. This is in agreement to the higher number of CBRs observed not to overlap with IDRs than the converse (Figure 2A,C). These results suggest that there is a significant number of CBRs that might not be disordered, whereas most disordered regions are compositionally biased.

Since, as discussed in the previous section, the properties of CBRs vary greatly with the type of amino acid, it is possible that differences in overlap with IDRs will also vary with type and may tell us something about the functional effects of composition bias in IDRs.

**Figure 3:**
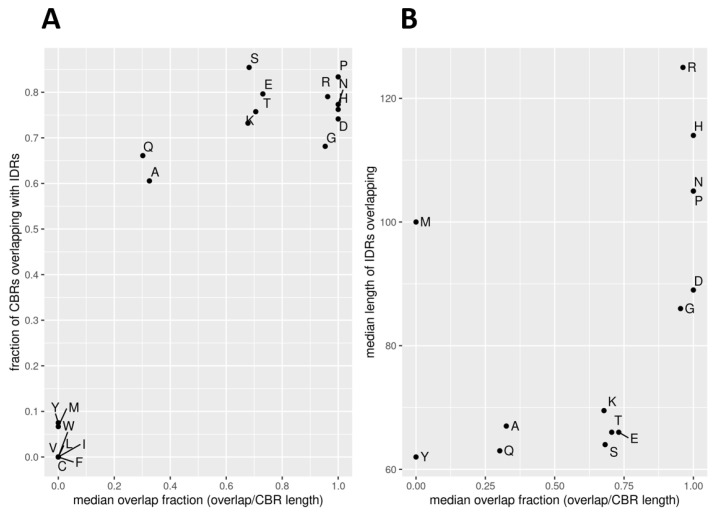
Extent of overlap of CBRs to IDRs by CBR type. (**A**) Fraction of CBRs that overlap any IDR (predicted with IUPred) versus the median fraction of the overlap relative to the total size of the CBR (0.5 means that half of the CBR overlaps an IDR). (**B**) Median length (residues) of overlapping IDRs versus the median fraction of the overlap relative to the total size of the CBR.

Indeed, we observe that many CBR types are not at all overlapping IDRs (Figure 3; Table 1). In fact, we observe that the frequency with which a particular type of CBR overlaps with predicted IDRs is either extremely low (zero for C, F, I, L, V and W, below 0.1 for M and Y) or relatively high (above 0.6). As commented above, these CBRs of hydrophobic residues have low occurrence and short lengths, even though some of these amino acids like L are very frequent. Together, these results indicate that CBRs of hydrophobic residues are unfavored, particularly in association with IDRs.

Analysis of the extent of coverage of the CBR by the IDR (in terms relative to the length of the CBR) further discriminates the types of frequently overlapping IDRs in three very distinct groups: overlap is full or close to full for P, G, R, D, H and N; around 0.7 for S, E, K and T; and around 0.3 for Q and A (Figure 3A). Again, this variation seems to be driven more by hydrophobicity and other physicochemical properties of the amino acids than by their frequencies. We note that the results from the MobiDB-lite IDR predictions are similar except for the T-CBRs that group with Q and A.

Consistently, all six CBR types with a high overlap length to IDRs also overlap with long IDRs (median length ≥ 86 residues; Figure 3B). All other types that overlap IDRs do so with shorter IDRs (median length in the range 50 to 70) with the exception of M-rich CBRs.

The CBR properties mentioned above, such as length or frequency, also do not seem to have much influence on the extent of the overlap. For example, D and R compose infrequent CBRs (below 500 occurrences; Figure 1A) but overlap often and strongly with IDRs (Figure 3A). We do observe that the group of CBRs from 8 amino acids that do not overlap IDRs result in very low numbers of CBRs (the most frequent being L with just 267 occurrences); their lengths could be defined as short but are not very different from those of CBR types that overlap IDRs. The rare M-rich CBRs stand out with the third largest average length (89 residues; Figure 1B) and also by their overlap to relatively long IDRs (median length 100; Figure 3B). CBR types with large median length do not necessarily overlap long IDRs (e.g., S- and Q-rich CBRs are the longest but overlap short IDRs; Figure 1B and Figure 3B, respectively).

These results indicate that CBRs of all types are possible, and that their properties of overlap with IDRs depend on the physicochemical properties of the contributing amino acid. We note that all CBR types that have no overlap to IDRs are extremely infrequent.

### 2.2. Position of the Overlap

**Figure 4:**
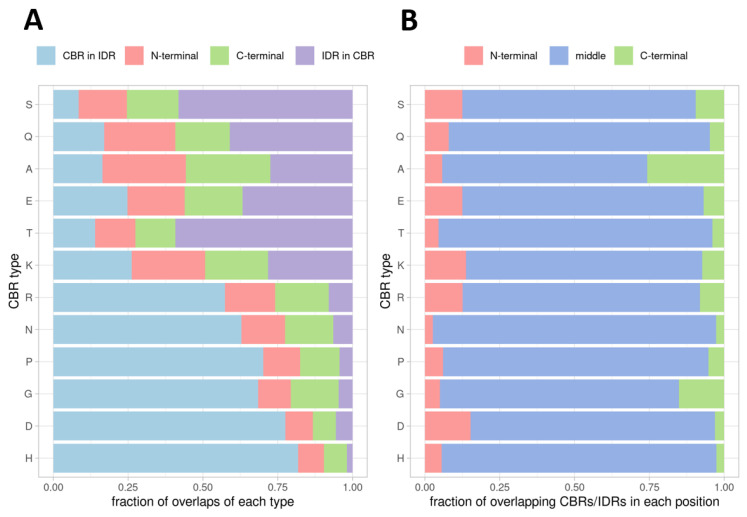
Position of CBR overlaps with IDRs. (**A**) Distribution of the positions of the overlaps of CBRs to IDRs (predicted with IUPred2A) relative to the IDR: IDR (included) in CBR, CBR (included) in IDR, CBR overlaps only the N-terminal of the IDR, CBR overlaps only the C-terminal of the IDR. (**B**) Distribution of the positions of overlaps of CBRs to IDRs by type relative to the protein: N-terminal 10 amino acids, C-terminal 10 amino acids, or else middle. CBR types have been arranged from longest (top) to shortest (bottom) median length (Table 1).

Next, we studied the relative position of CBRs and IDRs that overlap. We considered four cases: (i) the IDR is completely included in the CBR, (ii) the CBR is completely included in the IDR, or if they partially overlap, (iii) the CBR overlaps either the N-terminal of the IDR or (iv) the C-terminal of the IDR (Figure 4A). As it could be expected, there is a very good agreement with longer CBR types frequently containing entire IDRs (violet bars in Figure 4A; CBR types were arranged by median length from top to bottom) and shorter CBR types frequently being entirely inside IDRs (blue bars in Figure 4A). An exception could be T-rich CBRs: they overlap similarly short IDRs (median length in the 66–70 residue range) as A-, E- and K-rich CBRs, which also produce CBRs of similar median length (range 71–87 residues). Regardless, T-rich CBRs are the ones most-frequently fully containing IDRs and the second least frequently contained within IDRs (after S-rich CBRs, which have the longest median length, 159). Regarding the relative position of partially overlapping CBRs and IDRs, the frequency of N- and C-terminal overlaps for each type were very balanced, with G-rich CBRs showing the largest difference with more C-terminal overlaps.

CBRs and IDRs have been often noted in the termini of proteins, where it can be easily accommodated as they will interfere less with the globular parts, and display well-known regulatory functions such as the N-terminal of H1 histones (N-terminal tail domain, NTD) that sustain epigenetic regulatory patterns of acetylation and methylation [22] and interact with the C-terminal domain (CTD) of linker histones [23], the interactions of the disordered N-terminal domain (NTD) of the androgen receptor [24], or the disordered N-terminal transactivation domain and C-terminal tetramerization domain of p53 [25].

Accordingly, to try to identify particular types of CBRs associated with one or the other termini, we decided to explore the association of the overlaps of CBRs to IDRs to a terminal position in proteins. For this calculation, the position of an overlap between a CBR and an IDR was considered to be in the N- or C-terminal of the protein if either the CBR or the IDR overlapped the first or last 10 amino acids of the protein, respectively (Figure 4B). Here, we observed more asymmetry than in the relative overlaps (Figure 4A). D-rich CBRs had a high ratio of N- to C-terminal overlaps, while A- and G-rich CBRs were the ones with highest fractions of C-terminal overlaps.

### 2.3. IDRs and CBRs in the Human Protein Interaction Network

Interaction with proteins is one of the important functions that has been associated with IDRs [1]. We investigated if these interaction properties of IDRs are mediated by CBRs of particular types.

We hypothesized that among proteins with large numbers of interacting partners (also known as hub proteins) we might find some that will interact with a subset of partners using the same interface (mode of interaction). If this mode of interaction requires that the target protein has a particular type of CBR then this might be detectable because of its over-representation in the set of targets of the hub protein. We observed that this is the case for example for partners of human huntingtin with alanine rich regions in RASA1, SYN2 and KAT2B [26], which we took as a suggestion that such CBRs in those proteins might be involved in interactions with huntingtin.

We selected a set of hub proteins for study as those with 20 or more interactors from the HIPPIE database of scored protein interactions (8685 hubs [27]). We then studied the sets of proteins interacting with each hub to detect those significantly enriched in CBRs of particular types (which overlapped IDRs: IDR-CBRs) compared to the background of the proteome (*p*-value < 0.05; Fisher’s exact test). We repeated this analysis with a smaller set of hubs taking into account interactions of medium confidence or better (HIPPIE score ≥ 0.64; 7801 hubs) and then selected the IDR-CBRs whose enrichment improved in the strictest hub dataset (1902 IDR-CBRs and enrichments; Supplementary File S2).

Finally, to contrast the enriched CBRs with structural evidence for their participation in the interaction of IDRs with proteins, we looked for them in the database of disordered binding sites (DIBS [28]). Entries in this database include an experimental structure (from the Protein Data Bank) between a protein and a sequence fragment from another protein, which might be ordered in the complex but has been verified to be disordered in a different condition.

Using this approach, we obtained 153 pairs of hubs and interactors with IDR-CBRs with the interactors also being in DIBS (Table 2; Supplementary File S3). Among these, the most frequent types are P-, S- and E-rich regions, reflecting the frequency of CBRs in human proteins (Figure 1A). A stricter selection of CBRs overlapping the interacting fragments in DIBS resulted in 49 protein pairs, 19 of them S-rich CBRs (Table 2; Supplementary File S3), also not surprising considering that S-rich CBRs are the longest (Figure 1B). Q-, G- and K-rich CBRs were never found in the interface, except in one case (Table 2).

The 49 cases illustrate modes of protein interaction shared among proteins interacting with a hub-protein requiring a CBR within an IDR. Here, we present one case from each of the three frequently found types of CBRs.

S-rich CBR. The interaction of SUMO1 human (UniProt P63165) with an IDR in the E3 SUMO-protein ligase PIAS2 (UniProt O75928) has been structurally characterized [29] (DIBS DI1000007). The interacting IDR in PIAS2 is from position 466 to 490, which is harbored within a larger S-rich CBR from 437 to 613. The interacting IDR includes a SUMO binding amino acid sequence motif (SBM) (LIG_SUMO_SIM_par_1 in the ELM database [30]), which is by itself not serine rich. However, the S-rich CBR contains nearby known sites of serine phosphorylation reported in the UniProt entry, some of them in a tight cluster (e.g., at positions 476, 477 and 478).

It is expected that SUMO interacts with many proteins containing a SUMO interacting motif and the role of the S-rich region and their phosphorylated serines are probably necessary to control the partners allowed to compete for this protein hub.

P-rich CBR. The interaction of tumor susceptibility gene 101 protein (TSG101; UniProt Q99816) with a fragment of the Hepatocyte growth factor-regulated tyrosine kinase substrate (also known as Hrs, UniProt O14964) has been reported [31] (DIBS DI1000091). The interacting fragment (present in the protein from position 346 to 354: PTPSAPVPL) contains a four-residue motif (LIG_PTAP_UEV_1: PSAP); both are rich in prolines and are at the N-terminal of a larger P-rich CBR (from position 346 to 394). This motif binds to the UEV domain of Tsg101. The same work reports a very similar P-rich fragment (PEPTAPPEE) present in the HIV-1 Gag protein that binds similarly and is required for HIV-1 budding.

While there does not seem to exist other instances of the motif in Hrs, the presence of the P-rich region in these and other interactors of TSG101 seems to suggest their role in the interaction, may be forming other binding sites of lower affinity that might be used to guide the interaction.

E-rich CBR. MOB kinase activator 1A (Mob1; UniProt Q9H8S9) interacts with STK3 (Serine/threonine-protein kinase 3, also known as Mst2; UniProt Q13188). The interaction has been characterized (DIBS DI1000206) [32] and it happens via an IDR in Mst2 from positions 371–401, which overlaps a larger E-rich CBR from positions 293 to 376. Different to the cases above, the overlap with the CBR is partial: the C-terminal of the E-rich region overlaps the N-terminal of the interacting region. In fact, the E-rich overlap is not present in the solved structure suggesting that it remains flexible. Unlike the previous cases, in this case there is no predicted motif in the interacting IDR.

**Figure 5:**
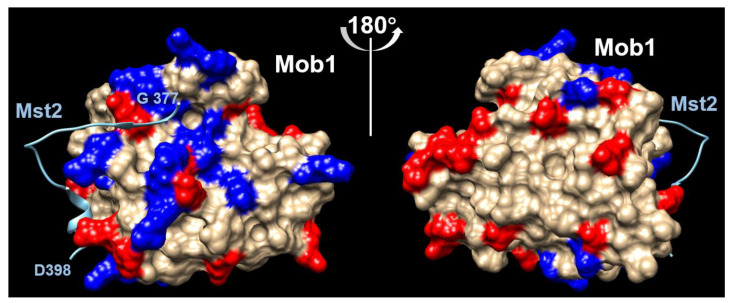
Interaction of Mob1 with Mst2. The blue ribbon represents a fragment of Mst2 interacting with Mob1. Positively (blue) and negatively (red) charged residues in the surface of Mob1 are indicated. Left and right show 180° rotated views of Mob1. The site in Mob1 surrounding the N-terminal of the interacting Mst2 fragment displays many positively charged residues, unlike the opposite side of the molecule. Using the modified PDB from the DIBS database (DI1000206 [28]).

Examination of the surface of Mob1 indicates a cluster of positively charged residues surrounding the N-terminal region of the Mst2 fragment (Figure 5). This suggests that the E-rich region, negatively charged, could work to aid non-specifically the localization of a sequence specific interaction. The finding that the interactors of Mob1 are enriched in E-rich CBRs suggests that this mechanism is used by other proteins to aid their specific interactions with Mob1, but the specific part of the interaction could be different to what we see for Mst2. In this case, the CBR region is near the binding region but does not take part directly on it.

### 2.4. IDRs-CBRs and the Cellular Environment

To find if IDR-CBRs could have properties for interaction with the cellular environment, we tested the differential presence of CBRs in drivers of liquid–liquid phase separation (LLPS) and in cellular locations depending on the enriched amino acid type and overlap to IDRs.

**Figure 6:**
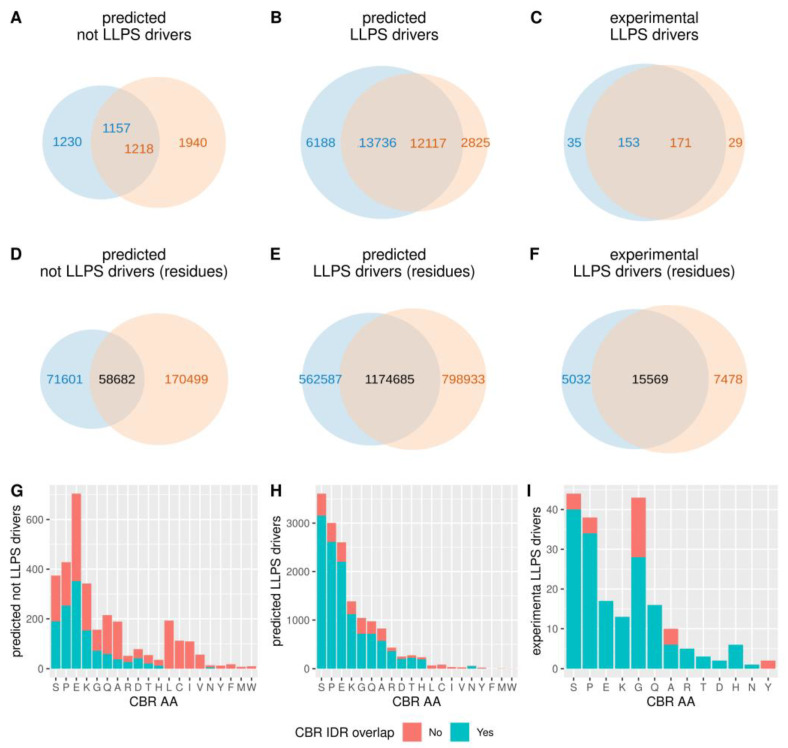
CBRs in LLPS drivers. Number of CBRs and their overlap to IDRs (predicted with IUPred2A) in human proteins predicted not to be LLPS drivers (**A**) or to be LLPS drivers (**B**) [34], or experimentally verified as LLPS drivers (**C**) (consolidated in the study of [33]) (see text and Methods for details). (**D**–**F**) Same as (**A**–**C**) but by number of residues. (**G**–**I**) show number of CBRs by type (ordered by global frequency) and their overlap to IDRs in predicted not LLPS drivers (**G**), predicted LLPS drivers (**H**) and experimental LLPS drivers (**I**).

As LLPS drivers we considered two datasets: one of 89 human LLPS drivers consolidated as sufficiently supported by physiologically relevant in vivo and in vitro experiments [33], and a much larger one of human proteins predicted to have the propensity to drive phase separation with the FuzDrop method [34]. From all human proteins, 40% are predicted as LLPS drivers (FuzDrop score > 0.5). Interestingly, they are enriched in CBRs and IDRs, as they contain 82% of all CBRs and 69% of all IDRs in the human proteome. Moreover, the CBRs and IDRs in predicted LLPS drivers overlap more compared to those in the rest of the proteins (81% of CBRs and 69% of IDRs in predicted LLPS, respectively, compared to 39% and 48% in other proteins, respectively; see Figure 6 A-B). In the dataset of experimentally verified LLPS drivers, the tendency of LLPS drivers to have more overlaps is also confirmed (Figure 6C). These tendencies are also maintained when computing the overlaps in terms of residues (Figure 6 D–F).

Regarding the type of CBRs, among the most frequent ones, we noted a higher frequency of E-rich CBRs and a lower frequency of S- and P-rich CBRs in proteins predicted as not LLPS drivers. Among the less frequent CBRs, L-, C-, I- and V-rich CBRs, which all do not overlap IDRs, have lower frequency in predicted LLPS drivers, and are absent from the set of experimentally verified LLPS drivers. Within experimentally verified LLPS drivers, the abundance of G-rich CBRs stands out as previously noticed (see, e.g., [35] and examples below).

Our findings are consistent with multiple reports of CBRs within the IDRs of LLPS drivers with a functional involvement in the LLPS process. One well characterized protein that shows this is RNA-binding protein FUS. FUS is disordered for almost all its length and G-rich for its first 500 residues. Glycine residues here enhance the fluidity [36] while the region is involved in phase separation [37]. DDX4, a probable ATP-dependent RNA helicase has an S-rich region overlapping closely with disorder in the first 200 residues and that is involved in phase separation [38]. TARDBP, TAR DNA-binding protein 43, has a G-rich region that overlaps with disorder in residues 273–413, around the same region being involved in phase separation [36].

**Figure 7:**
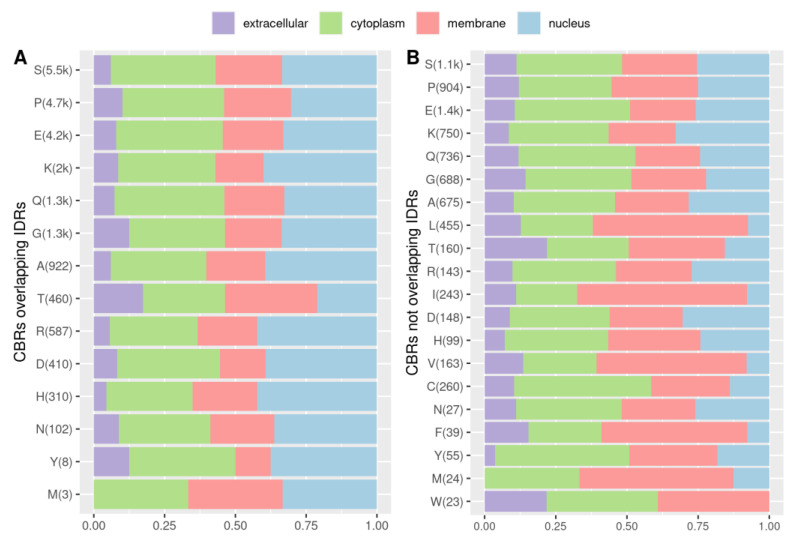
CBRs in cellular locations. (**A**) CBRs overlapping IDRs. (**B**) CBRs not overlapping IDRs. Locations were obtained from Gene Ontology annotations retrieved with QuickGO (see Methods for details). Number of CBRs for each category is indicated in brackets (k means thousands).

Comparing the subcellular location of the proteins that contain CBRs, overlapping or not overlapping with IDRs, we could notice certain differences. We used four different categories of location based on Gene Ontology terms: cytoplasm (based on the term “cytoplasm”), extracellular (term “extracellular region”), membrane (term “membrane”) and nuclear (term “nucleus”) (see Methods for details). We found 18,222 such annotations, counting multiple annotations for the same proteins, and 13,530 of those were for proteins with overlaps of IDRs and CBRs. CBRs overlapping IDRs are significantly more often found in proteins located in the nucleus, and less in the membrane and in the extracellular region compared to CBRs not overlapping IDRs (Fisher’s exact test *p*-value < 0.05; Figure 7).

## 3. Conclusions

We found that the majority of IDRs and CBRs overlap (Figure 2) and that the extent of overlap of a CBR type depends more on the physicochemical properties of the enriched amino acid (mostly polarity) than on their background frequencies (Figure 3). Because all CBR types that have no overlap to IDRs have low frequency (Figure 3A), we hypothesize that there is selection pressure against those CBR types. Being placed inside a disordered region seems to be the function CBRs are selected for.

We found very little if none positional biases of CBRs within IDRs and proteins. This agrees with the fundamentally unstructured quality of IDRs. Without globular fixed coordinates, the positions of amino acids in space lack relevance and only their relative distances matter at short ranges as in motifs (e.g., SLiMs). For example, G-rich CBRs within IDRs including a few arginines have been denominated GAR domains and RGG motifs and are present in nucleolar proteins where they aid interaction with RNA: in the N-terminal of Fibrillarin [39], in the C-terminal of nucleolin [40], or in both terminals in GAR1 [41].

Our studies of IDR-CBRs suggest their functionality in the context of cellular interactions and organization. Considering the IDR-CBRs more enriched in sets of hub interactors (Table 2), S- and P-rich CBRs seem to reflect the generation of tandem motifs for cooperative regulation [4], while E-rich CBRs could result in electrostatic non-specific interactions, and the conspicuous absence of G-, Q- and K-rich IDR-CBRs would suggest that they are not used to promote IDR interactivity.

We observed that most IDRs and CBRs are present in proteins predicted to be phase separation drivers and that they overlap more often between each other in that case comparatively to when they are not in such proteins (Figure 6), suggesting that IDR-CBRs have a role in LLPS, for which there are already well-known examples (e.g., FUS, DDX4, TARDBP). We also found that the nucleus is enriched in proteins with higher overlap of CBRs and IDRs (Figure 7); the nucleus holds proteins with many interactions such as transcription factors and epigenetic regulators and as a result protein–protein interaction networks in the nucleus are denser and include more hubs compared to other regions [42].

Overall, our results, specific for human proteins, suggest that (i) the overlap of CBRs and IDRs is significant, (ii) their position within proteins and relative to each other is not strongly associated with function, (iii) the most prominent functions that can be assigned have to do with the organization of cellular compartments and protein interactions, and (iv) we could not associate CBR types to specific functional mechanisms, save for the existence of S- and P-rich motifs in interactions.

We observed that the results and conclusions produced were independent of the disorder prediction tool used, between IUPred2A and MobiDB-lite, although the number of regions and residues predicted as disordered was not the same.

One important limitation of our study is that we necessarily simplified our analysis by considering all CBRs enriched in a particular amino acid type as a group. In reality, the non-enriched amino acids might play a role in the properties of the CBR, particularly when the enriched amino acid is a non-charged type of amino acid like G, A, S or T (as in the case of G-rich regions with RGG motifs [43]). Studying regions rich in two or a few amino acids is a reasonable extension of our work.

The extent and concentration in particular protein types that we found for the overlap between CBRs and IDRs demonstrate that composition bias is dynamically and specifically selected within IDRs in human proteins. Complementary studies in other species and contrasting the functional equivalence of orthologs from species at various evolutionary distances should increase our knowledge about the evolution and function of IDRs, which we need to complete our understanding of protein interactions and their regulation.

## 4. Methods

Our protein set were the 20,609 proteins from the 01.2021 UniProt reference proteome. For the prediction of CBRs, we used one of the few tools available for this purpose, CAST v2.0 [5], which uses a Smith-Waterman comparison of the query sequence against twenty homopolymers. The CBRs found by CAST can be of any length, and in our data set they vary from 5 to 12,000 residues (UniProt Q8WXI7, Mucin-16, has the two largest CBRs). For the IDR prediction there are a plethora of tools that are created and evaluated by the community every year, such as in the Critical assessment of protein intrinsic disorder (CAID) experiment [44]. We chose to use IUPred2a and the readily online available predictions of MobiDB-lite as these perform well in the experiment mentioned above and are easy to use.

We ran CAST v2.0 with the default parameters and IUPred2a with the “long” setting that looks for IDRs of a minimum length of 30 amino acids. We downloaded the MobiDB-lite disorder predictions from the MobiDB database [20,21], and used those for the same 20,609 proteins of the reference proteome.

The Gene Ontology annotations were retrieved with QuickGO selecting terms GO:0016020 (membrane), GO:0005576 (extracellular region), GO:0005737 (cytoplasm), GO:0005634 (nucleus), selecting “Use these terms as a GO slim” and including “is_a”, “part_of”, “occurs_in” relations.

For each CBR found in each protein we reported the overlap to any IDRs and we also categorized the overlaps in two ways: in regard to the position of the overlap in the protein (N-terminal, C-terminal, middle) and in regard to the position of the IDR in relation to the CBR (N-terminal of the CBR, C-terminal, IDR in CBR, CBR in IDR). The position of the overlap or the IDR was considered to be in the N or C terminal if it overlapped the first or last 10 amino acids, respectively.

We obtained a dataset of predicted LLPS drivers (using the FuzDrop method; score > 0.5; Supplementary Table S7 in [34]). In that work, FuzDrop was run on a version of the SwissProt human proteome with 20,367 proteins. In addition, we obtained a dataset of experimentally verified LLPS drivers from Supplementary Table S2 in [33].

The protein hubs we used are proteins with 20 or more interactions we recovered from the HIPPIE database of scored protein interactions [27] (8685 hubs). We used a Fisher’s exact test to detect the interactors of each protein hub that were enriched in CBR-IDR overlaps compared to the background of the proteome (*p*-value < 0.05). We repeated this analysis with a smaller set of hubs only including interactions of medium confidence or better (HIPPIE score >= 0.64; 7801 hubs) and then selected the IDR-CBRs whose enrichment improved in the strictest hub dataset (1902 IDR-CBRs and enrichments; Supplementary File S2). We also recovered the same protein hubs that were present in the database of disordered binding sites (DIBS [28]).

The figures were produced with R and packages ggplot2 and VennDiagram.

## Figures and Tables

**Table 1:** Properties of CBRs by type. Columns indicate type, number of CBRs, total of residues, median length, fraction of overlap to IDRs in numbers of regions and in residues, and general frequency of the amino acid in the human proteome (shown for comparison). Note that CBRs of different types might overlap and thus the total number of residues in this table is higher than the number of residues in CBRs (2,597,987 and 2,215,396, respectively).

Type	Number	Total Residues	Median Length	Fraction Regions Overlap IDRs	Fraction Residues Overlap IDRs	aa Frequency
S	3997	1,026,360	159	0.84	0.63	0.083
P	3459	233,649	40	0.83	0.88	0.063
E	3328	506,115	83	0.77	0.53	0.071
K	1734	186,315	71	0.74	0.58	0.057
G	1207	88,628	31	0.66	0.77	0.066
Q	1195	229,995	112	0.65	0.33	0.048
A	1021	123,263	87	0.6	0.43	0.070
R	494	42,303	58.5	0.79	0.73	0.056
T	329	89,424	81	0.74	0.69	0.054
D	327	13,609	27	0.73	0.77	0.047
H	268	14,252	23	0.75	0.87	0.026
L	267	10,331	26	0	0	0.100
C	199	15,837	46	0	0	0.023
I	145	4885	25	0	0	0.043
V	86	3985	27	0	0	0.060
N	75	4740	44	0.77	0.78	0.036
Y	40	1921	41.5	0.05	0.02	0.027
F	20	641	28	0	0	0.037
M	16	1572	89	0.06	0.05	0.021
W	15	162	10	0	0	0.012

**Table 2:** IDR-CBRs in protein hubs and their interaction interfaces. Columns indicate: CBR type, how many interactors with this particular type of IDR-CBRs were found (i) enriched in sets of interactors of hub proteins, (ii) of those how many were in sets whose enrichment improved when selecting higher quality interactions, (iii) of those how many were present in an entry considering their interaction with the hub protein in the DIB database, and (iv) of those in how many the enriched IDR-CBR overlapped with the defined region interacting with the hub protein. Details are available in Supplementary Files S2 and S3.

CBR Type	Enriched	Improved	DIBS	Interface
E	78,842	3601	27	11
S	73,436	5325	27	19
K	46,014	1909	10	0
P	37,150	3721	58	16
Q	20,112	1257	10	1
G	16,207	1107	11	0
R	10,449	611	0	0
A	8500	561	4	1
D	6616	273	3	1
H	1776	89	1	0
T	1760	181	2	0
N	606	30	0	0

## Data Availability

All relevant supporting data is provided as Supplementary files.

## Supplementary Materials

The following supporting information can be downloaded at: https://www.mdpi.com/article/10.3390/biom12101486/s1: Supplementary File S1. CBRs and IDRs detected in human proteins. Protein sequence (UniProt ID), features and coordinates. Supplementary File S2. Hubs and enriched IDR-CBRs in their interactors. The columns indicate, hub protein, enriched CBR type in its interactors, *p*-value of the enrichment considering all PPI data (pval1), *p*-value considering good quality PPI data (pval2), list of CBR containing proteins interacting with the hub protein. Supplementary File S3. IDR-CBRs in interaction hubs and interacting interfaces. The columns indicate, hub protein, CBR type, interactor protein, identifier of the DIBS entry describing the interaction, start and end of the IDR interacting fragment in DIBS, start and end of the CBR, and overlap (TRUE or FALSE).
